# Diversity and behavioral activity of *Anopheles* mosquitoes on the slopes of Mount Cameroon

**DOI:** 10.1186/s13071-022-05472-8

**Published:** 2022-09-28

**Authors:** Pilate N. Kwi, Elvis E. Ewane, Marcel N. Moyeh, Livinus N. Tangi, Vincent N. Ntui, Francis Zeukeng, Denis D. Sofeu-Feugaing, Eric A. Achidi, Fidelis Cho-Ngwa, Alfred Amambua-Ngwa, Jude D. Bigoga, Tobias O. Apinjoh

**Affiliations:** 1 Department of Biochemistry and Molecular Biology, University of Buea, Buea, Cameroon; 2 Department of Medical Laboratory Sciences, University of Buea, Buea, Cameroon; 3 Department of Chemical and Biological Engineering, The University of Bamenda, Bamenda, Cameroon; 4 Department of Microbiology and Parasitology, University of Buea, Buea, Cameroon; 5 Laboratory for Vector Biology and Control, The Biotechnology Centre, University of Yaounde 1, Yaounde, Cameroon; 6 Medical Research Council Unit The Gambia at London, School of Hygiene and Tropical Medicine, Fajara, The Gambia

**Keywords:** Malaria, *Anopheles*, Diversity, Altitude, Infectivity

## Abstract

**Background:**

Malaria remains endemic in Cameroon, with heterogeneous transmission related to eco-climatic variations, vector diversity and spatial distribution. The intensification of malaria prevention and control through the free distribution of insecticide-treated nets in recent years may have altered the composition, geographic distribution and natural infection rate of *Anopheles* species, with implications for malaria transmission dynamics. The present study seeks to assess the vectorial diversity, dynamics and infectivity across different seasons and altitudes in relationship to parasite prevalence around the slopes of Mount Cameroon, southwestern region.

**Method:**

Mosquitoes were sampled (indoors and outdoors) in 11 eco-epidemiological settings at low (18–197 m), intermediate (371–584 m) and high (740–1067 m) altitude by nightly human landing catches. The mosquitoes were identified morphologically and *Anopheles gambiae* sibling species identified by PCR. Parity status was ascertained by examining the ovaries and the entomological inoculation rates (EIR) determined by *Plasmodium falciparum* circumsporozoite antigen ELISA of the head-thorax. The prevalence of *Plasmodium* infection across target communities was assessed using rapid diagnostic tests.

**Results:**

A total of 7327 (18.0 mosquitoes/trap/night) mosquitoes were trapped, mainly during the rainy season (5678, 77.5%) and at low altitude (3669, 50.1%). *Anopheles* spp. (5079, 69.3%) was the most abundant genera and *An. gambiae* complex (2691, 36.7%) the major vector, varying with altitude (*χ*^2^ = 183.87, *df* = 8, *P* < 0.001) and season (*χ*^2^ = 28.14, *df* = 4, *P* < 0.001). Only *An. gambiae* (s.s.) was identified following molecular analysis of *An. gambiae* complex siblings. The overall biting peak for *An. gambiae* complex was 2—3 a.m. *Anopheles cinctus* was the most abundant secondary vector in the area. The average EIR in the area was 2.08 infective bites per person per night (ib/p/n), higher at low (2.45 ib/p/n) than at intermediate altitude (1.39 ib/p/n) and during the rainy (1.76 ib/p/n) compared to the dry season (0.34 ib/p/n). *Anopheles funestus* was most infectious overall (28.1%, 16/57) while *An. gambiae* had the highest inoculation rates averaging 1.33 ib/p/n. Most *Anopheles* species across all altitudes and seasons were parous, highest in communities with the highest proportion of malaria parasite infections.

**Conclusion:**

*Anopheles gambiae* (s.s.) remains the major malaria vector in the area and *An. cinctus* possibly a secondary vector of the disease in the slopes of Mt. Cameroon. The seasonal and altitudinal effects on the distribution of these mosquitoes may have implications for the transmission of malaria and its control strategies in the area. Regular monitoring of the bionomics of local *Anopheles* vector species and targeted control interventions in the ‘hotspots’ is necessary to curb the prevalence of the infection and incidence of disease.

## Background

Malaria remains a significant global health concern, especially in children < 5 years in sub-Saharan Africa (SSA), where 95% of cases and 96% of the deaths occur [1]. The disease is transmitted to people through the bites of infected female *Anopheles* mosquito, with 70 of the approximately 490 *Anopheles* species known to be malaria vectors [2]. In SSA, approximately 20 of the 140 *Anopheles* species are known to transmit malaria parasites to humans [3]. Of these, *Anopheles gambiae* sensu stricto (s.s.), *An. gambiae* sensu lato (s.l.), *An. arabiensis, An. funestus* group and *An. nili* group are the most widely distributed and important vector species in tropical Africa [2–5]. Furthermore, only 18 of these 140 have been documented and incriminated for malaria transmission in Cameroon [3, 6–8]. *Anopheles arabiensis, An. coluzzii, An. gambiae* and *An. funestus* (s.s.) are classified as primary vector species for *Plamodium* spp. transmission in Cameroon while *An. carnevalei, An. coustani, An. hancocki, An. leesoni, An. marshallii, An. melas, An. moucheti, An. nili, An. paludis, An. pharoensis, An. ovengensis, An. rivulorum-like, An. wellcomei* and *An. ziemanni* play a secondary role [9]. All these species display strong anthropophilic host-seeking behavior and longevity, causing many malaria cases [1].

Identifying vector species in different ecological zones is essential for the planning and implementation of vector control measures. In addition, assessments of malaria parasite transmission dynamics and the risk of human infection as well as the interaction among humans, parasite and vectors are also imperative. This depends on several factors, including the human biting rate (the frequency at which a human is exposed to mosquito bites), proportion of biting mosquitoes that are infectious, altitude, topography, hydrology and landscape occupied by humans and/or peridomestic animals [10–12]. Thus, the abundance of anopheline mosquito species is the most common entomological measure for determining the relationship between vectors and malaria incidence in any locality [13, 14].

Although a number of alternative sampling methods exist [15], human landing catches (HLCs) remains the most appropriate method for information about the actual behavioral activities of mosquitoes [16]. This is still the gold standard for measuring exposure of humans to mosquito bites [16] and estimating the human biting rate (HBR), a key determinant of the entomological inoculation rate (EIR) and a measure of malaria transmission [17]. In Cameroon, like in other parts of Africa, malaria transmission is very heterogeneous because of eco-climatic variations, seasonality, rainfall and precipitation [18]. Malaria transmission is seasonal in most parts of Cameroon, hyperendemic and hypoendemic during the rainy and dry season respectively around the slopes of Mount Cameroon in the southwestern region [18].

Malaria control hinges on vector control through the use of long-lasting insecticide-treated nets (LLINs) and indoor residual spraying (IRS). The concomitant reduction in malaria mortality following the scale-up in ITN coverage prompted the government of Cameroon, with support from Global Fund for the Fight against HIV, Malaria, and Tuberculosis, to distribute millions of LLINs to most households in the country, including the southwestern region, in line with the Roll Back Malaria (RBM) recommendation of universal coverage [19]. Mass and free distributions of LLINs remain the main national malaria vector control intervention strategy [20] and have been shown to effectively reduce the prevalence of the disease in the area [21] and country [18] and perhaps the vector population and their dynamics. In addition, the state sanitation company, Hysacam, contracted since 2011 to dispose of garbage and improve community environmental hygiene [22], has faced serious challenges in workflow over the last few years. This is due partly to the sociopolitical crisis that has affected the region since 2016, with implications for mosquito-breeding sites, anopheline density and malaria parasite transmission.

Potential reductions of the malaria burden in endemic and epidemic regions depend on knowledge of the malaria-transmitting mosquito species, populations behavioral characteristics and malaria exposure risks [6]. However, data on the susceptibility of *Anopheles* mosquitoes in the southwest region of Cameroon, home to the largest agro-industrial complex in the Central African Sub-Region, remain limited [9, 23, 24] and may not reflect the current dynamics. In addition, little is known about their seasonal and altitudinal distribution of the anopheline fauna in the area despite their documented impact on parasite prevalence [25]. The present study seeks to assess the vectorial diversity, dynamics and infectivity across different seasons and altitudes in relationship to parasite prevalence around the slopes of Mount Cameroon, southwestern region.

## Methods

### Study area

**Fig. 1:**
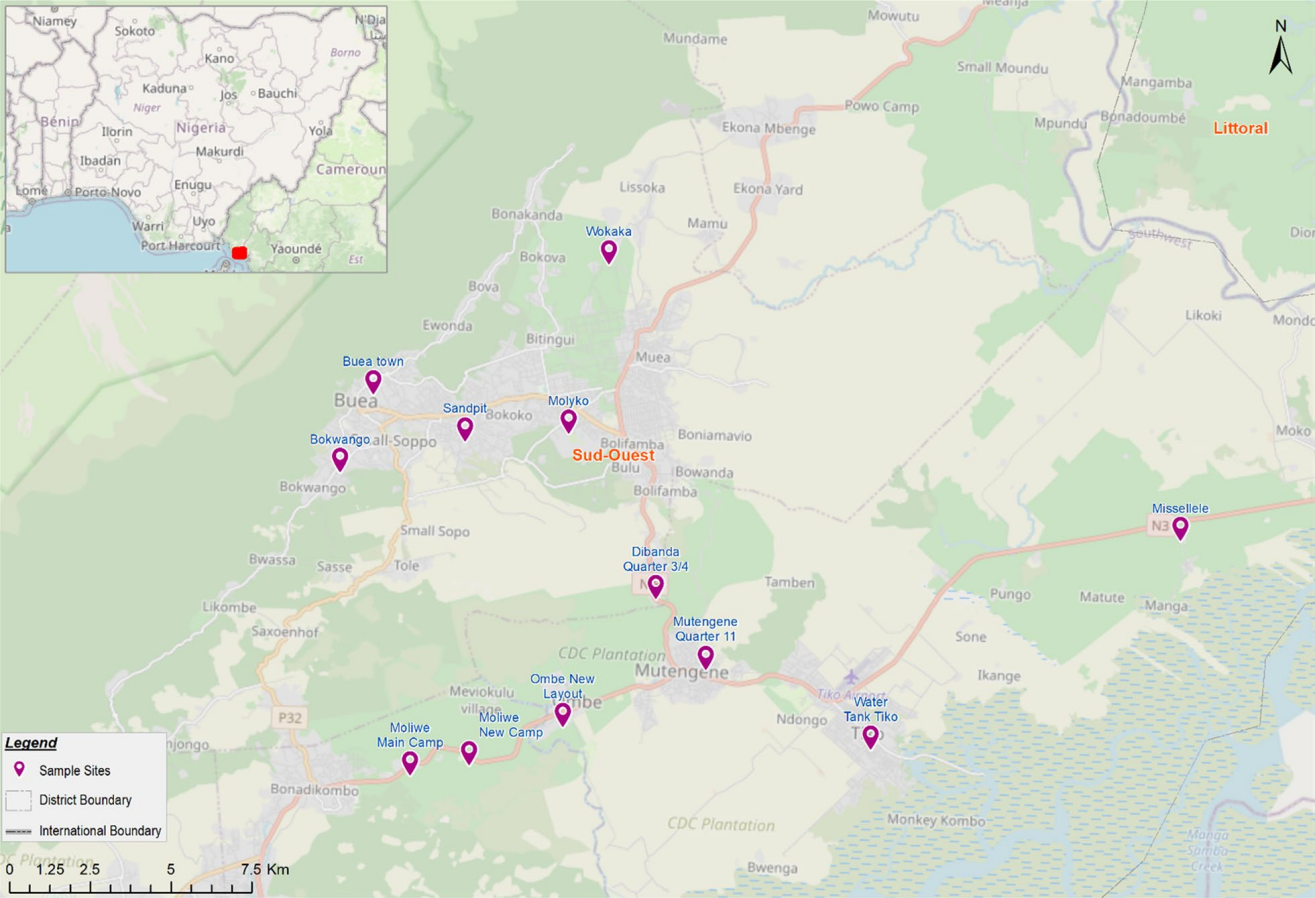
Study area and sampling sites

The study was carried out in the Buea, Limbe and Tiko health districts along the slopes of Mount Cameroon in the southwest region of Cameroon in selected eco-epidemiological settings at low (18–197 m), intermediate (371–584 m) and high (740–1067 m) altitude (Fig. 1). This forested region, located close to the Atlantic Ocean, is made up of low land and hilly areas, consisting of villages and towns, some of which are close to water bodies such as streams, ponds and rivers [24]. The southwest region of Cameroon has Buea as its capital, and as of 2015, its population was estimated to be 1,553,320. The area has an equatorial climate characterized by daily temperatures ranging between 20–33 °C, average annual rainfall of 2625 mm, high relative humidity (75–80%) and precipitation (2000–10,000 mm) [24] and two major seasons, a long rainy season (March–November) and a short dry season (November–February) [26], although the pattern is changing. Houses in the areas are made of plank/wood, mudbrick or cement blocks with aluminum metal sheets. Livestock like dogs, goats and fowl are the most common peridomestic animals and agriculture is the main occupation.

### Study design and sites

This was a cross-sectional study carried out between April 2020 and September 2021. Following malaria parasite prevalence surveys, 11 communities across different altitudinal zones [low (18—197 m), intermediate (371—584 m) and high altitude (740—1067 m)] and localities (rural, semi-urban and urban) were selected (Table 1, Fig. 1) [21, 24], each community at least 5 km from the next. Mosquitoes were trapped at 68 randomly selected sites across all altitudes during the rainy and dry seasons. A total of 172 malaria rapid diagnostic test (mRDT)-negative collectors and 68 supervisors were involved in the study, with an average distance of ∼ 500 m maintained between the collection sites in every community.

**Table 1:** Geographic characteristics of the study sites in the Mount Cameroon area

Community	Geographic centroids	Altitude		Locality
		Masl	Class	
Missellele	4°07′50ʺ N, 9°26′36ʺ E	18	Low	Rural and Semi-urban
Tiko	4°04′28ʺ N, 9°21′57ʺ E	24		
Ombe	4°04′40ʺ N, 9°17′13ʺ E	173		
	4°04′02ʺ N, 9°15′48ʺ E	173		
Moliwe	4°03′51ʺ N, 9°14′54ʺ E	187		
Mutengene	4°05′37ʺ N, 9°19′23ʺ E	197		
Dibanda	4°06′43ʺ N, 9°18′35ʺ E	371	Intermediate	
Wokaka	4°11′49ʺ N, 9°17′42ʺ E	488		Rural
Molyko	4°09′11ʺ N, 9°17′10ʺ E	584		Urban
Sandpit	4°09′01ʺ N, 9°15′35ʺ E	740	High	Semi-urban
Bokwango	4°08′29ʺ N, 9°13′41ʺ E	978		
Buea Town	4°09′42ʺ N, 9°14′09ʺ E	1067		

*Masl* meters above sea level

### Malaria rapid diagnostic testing

The malaria parasite prevalence of selected communities was determined by screening volunteer individuals by malaria rapid diagnostic test (mRDT) of the capillary blood using the *PfHRP2*/pLDH mRDT kit (SD Bioline, Alere, South Korea) and according to the manufacturer’s instructions. In addition to the community-wide cross-sectional survey, malaria tests were also done on prospective mosquito catchers, as described above, and all malaria-positive potential catchers excluded from the study.

### Household survey

For mosquito sampling, each community was divided into 4 different sectors, each of which had a minimum of 20 houses. One house was then randomly selected from each sector for mosquito collection, subject to the consent of the household head, failing which, a neighboring household was chosen.

### Mosquito sampling and identification

Mosquito collections were undertaken in most communities immediately following malaria prevalence survey. Mosquitoes were trapped monthly for 3 consecutive nights, both indoors and outdoors, by human landing catches (HLC), each collection period lasting for 12 h uninterrupted, from 6:00 p.m. to 6:00 a.m. To increase the effectiveness of the trapping and avoid catchers falling asleep, four catchers were used at every sector, two between 6:00 p.m.–12:00 a.m. and the other two from 12:00 a.m. to 6:00 a.m. The catchers used mouth aspirators, flashlights and stopwatches to trap mosquitoes from their exposed legs. To reduce bias due to individual differences in skill and attraction to mosquitoes, catchers were rotated between shifts and days of collection. The mosquitoes were then sorted by genus and the anophelines identified morphologically under a stereomicroscope (Olympus SZX10, Berlin, Germany) using the WHO pictorial identification key of important disease vectors [27] and the keys of Maureen Coetzee [28]. The parity status of a proportion of unfed mosquitoes was ascertain by dissecting their ovaries and examining the tracheoles as described previously [29]. This indicates the proportion of older mosquitoes within the population during the survey and is used as a proxy measure for the daily survival rate and average life span of the vector population.

### Molecular identification of *An. gambiae* complex

A total of 160 mosquitoes of the *An. gambiae* complex were randomly selected (40 each per season at both the low and intermediate altitudes) for molecular speciation. Briefly, genomic DNA was extracted from the legs/abdomen of the mosquitoes using the DNAzol protocol as described [6]. The DNA was re-suspended in 10 μl sterile TE buffer (10 mM Tris–HCl pH 8.1, 1 mM EDTA) and the *An. gambiae* sibling species identified by multiplex PCR using predesigned ribosomal DNA-specific primers as described previously [30]. PCR products were subsequently separated on 2% agarose in TBE containing ethidium bromide at 10 mg/ml and visualized on a UV illuminator (TOYOBO Trans Modele TM-20) against a 1-kb ladder.

### Estimation of entomological parameters

The head-thorax segment of a total of 313 randomly selected *Anopheles* species, including the 160 mosquitoes of the *An. gambiae* complex molecularly identified above, 51 each of *An. funestus*, *An. nili* and *An. hancoki* were tested for the *P. falciparum* circumsporozoite antigen by ELISA as described [31]. In each assay, test samples giving optical densities (OD) that exceeded the corresponding mean + 2 standard deviations for 4 negative control samples (male *Anopheles* mosquito) were considered seropositive.

### Data analysis

All data were entered into Microsoft Excel 2019 and analyzed using SPSS Statistics 24 for windows (IBM, CA, USA). Associations between normally distributed qualitative parameters were assessed using the chi-square test while differences in group means were assessed using the Student *t* test or analysis of variance (ANOVA). Statistical significance level was set at *P* < 0.05.

The four main measures of malaria vector transmission include; human biting rate (HBR), sporozoite index (SI), parity rate (PR) and entomological inoculation rate (EIR) as stated previously [6].

- HBR = number of mosquitoes caught per person per night of sampling.
- SI = the proportion of anophelines with *P. falciparum* circumsporozoite antigen = (ELISA positive samples/total sample analyzed by ELISA) × 100.
- PR = number of female anophelines that laid eggs at least once (parous) multiplied by 100 and divided by the total number of females dissected.
- EIR, a key variable in expressing malaria transmission levels, was calculated as the product of anopheline-biting rate and SI.

## Results

### Mosquito density and diversity

**Fig. 2:**
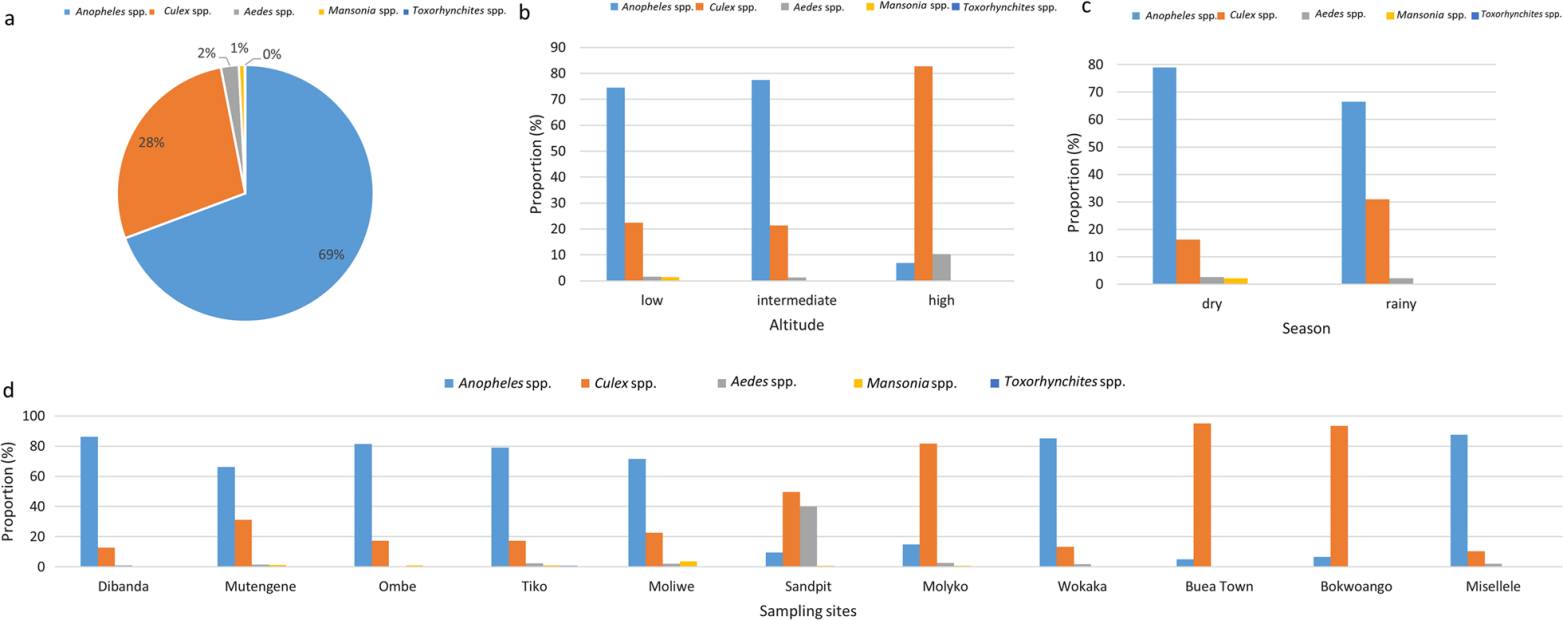
Distribution of the genera of mosquitoes around the slopes of Mount Cameroon. **a** Overall distribution in the survey. **b** Variation across altitude. **c** Variation across seasons. **d** Variation across sampling communities

A total of 7327 (18.0 mosquitoes/trap/night) mosquitoes were trapped during the entire survey period, mainly during the rainy season (5678, 77.5%) and at low altitude (3669, 50.1%). Five genera were recorded (Fig. 2): *Anopheles* spp. (5079, 69.3%), *Culex* spp. (2024, 27.6%), *Aedes* spp. (165, 2.3%), *Mansonia* spp. (55, 0.8%) and *Toxorhynchites* spp. (4, 0.1%). There was a significant variation in mosquito diversity across the sampling communities (*χ*^2^ = 3557.91, *df* = 40, *P* < 0.001), altitudes (*χ*^2^ = 1535.87, *df* = 8, *P* < 0.001), and season (*χ*^2^ = 191.68, *df* = 4, *P* < 0.001), with *Anopheles* spp. most abundant in Misselele (87.4%, 298/341) and Dibanda (86.3%, 1952/2262) at intermediate altitude (77.4%, 2297/2969) and during the dry season (78.9%, 1301/1649). Nevertheless, *Culex* spp., the majority of which were *Culex pipiens* (71.7%, 1451/2024), dominated the mosquito fauna in Buea Town (95.1%, 196/206) and Bokwoango (92.3%, 286/306) at high altitude (82.7%, 570/689) and during the rainy season (30.9%, 1756/5678). Most *Aedes* mosquitoes were caught from sandpit (40.1%, 71/177) while all (100%, 4/4) *Toxorhynchites* spp. were caught from Tiko at low altitude during the rainy season. In all, very few anophelines were trapped at high altitude (6.8%, 47/689) compared to intermediate (77.4%, 2298/2969) and low (74.5%, 2735/3669) altitudes.

### Anopheline species diversity and biting density

**Fig. 3:**
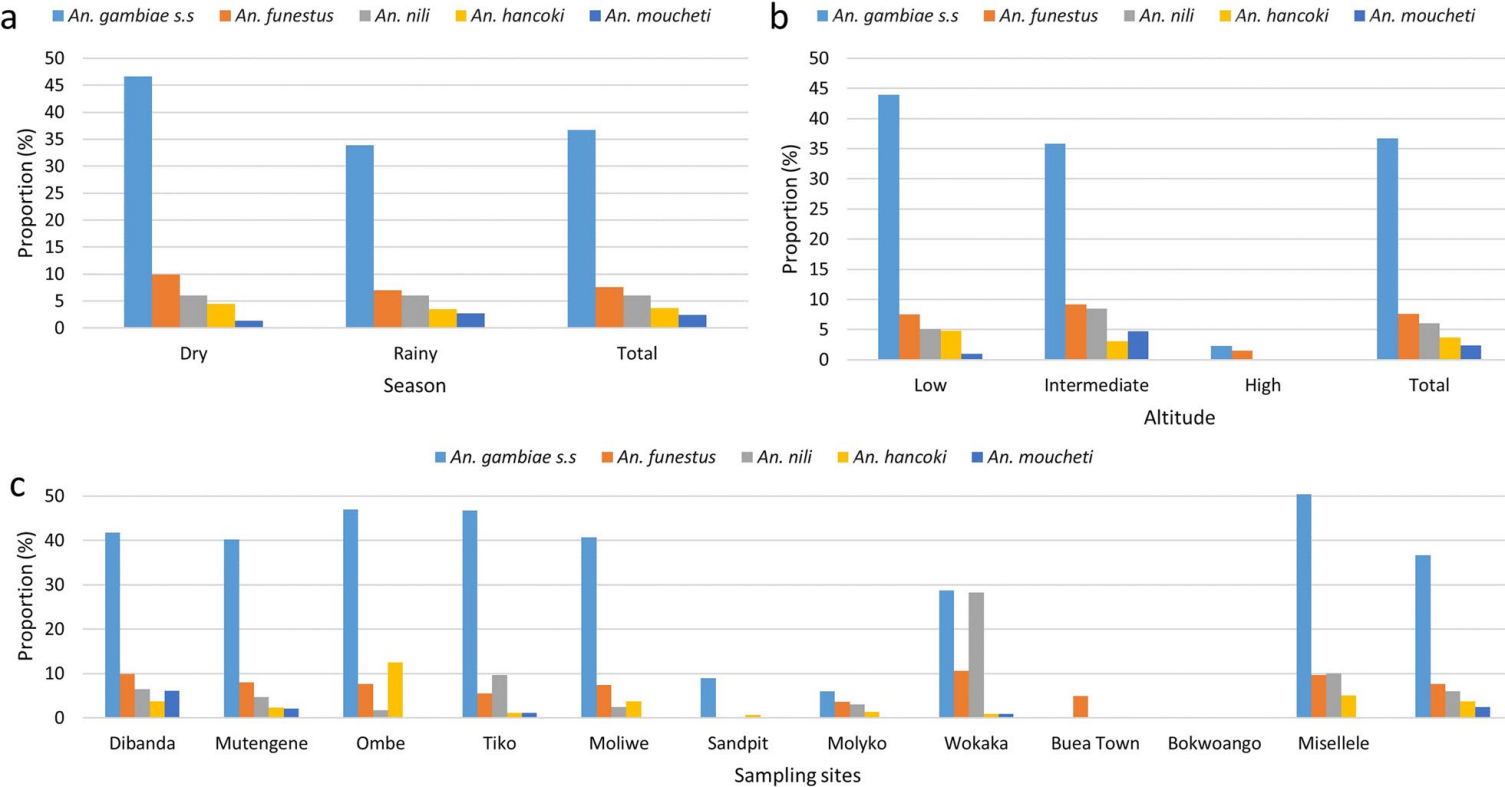
Variation in major malaria vector populations across the slope of Mount Cameroon. **a** Variation across seasons. **b** Variation across altitude. **c** Variation across sampling communities

A total of 5079 anophelines were trapped, giving an average density of 12.4 *Anopheles* mosquitoes/trap/night across all sites. In all, 81.4% (4136/5079) of the *Anopheles* spp. fauna were major malaria vectors. *Anopheles gambiae* complex (2691, 36.7%), *An. funestus* (560, 7.6%), *An. nili* (438, 6.0%), *An. hancoki* (270, 3.7%) and *An. moucheti* (177, 2.4%) distribution (Fig. 3) varied significantly with altitude (*χ*^2^ = 183.87, *df* = 8, *P* < 0.001) and season (*χ*^2^ = 28.14, *df* = 4, *P* < 0.001). Interestingly, the proportion of all major vectors, except *An. moucheti,* was higher in the dry season compared to the wet season. The density of *An. gambiae* complex was highest at low altitude, *An. funestus* group and *An. nili* complex at intermediate altitude while anophelines were almost nonexistent, overall at high altitude.

Molecular analysis of *Anopheles gambiae* complex siblings revealed only the presence of *An. gambiae* (s.s.). Furthermore, *An. cinctus* (155), *An. longipalpis* (117), *An. kingi* (106), *An. ziemanni* (100) and *An. marshalli* (67) are proposed as secondary vectors of malaria in the area, constituting up to 10.7% (545/5079) of the population of anophelines caught. This is the first report of *An. cinctus* as a possible secondary malaria vector in the area. Some 173 (2.4%) of the anophelines did not match the characteristics of any known species according to the manual.

### Anopheles biting behavior

**Fig. 4:**
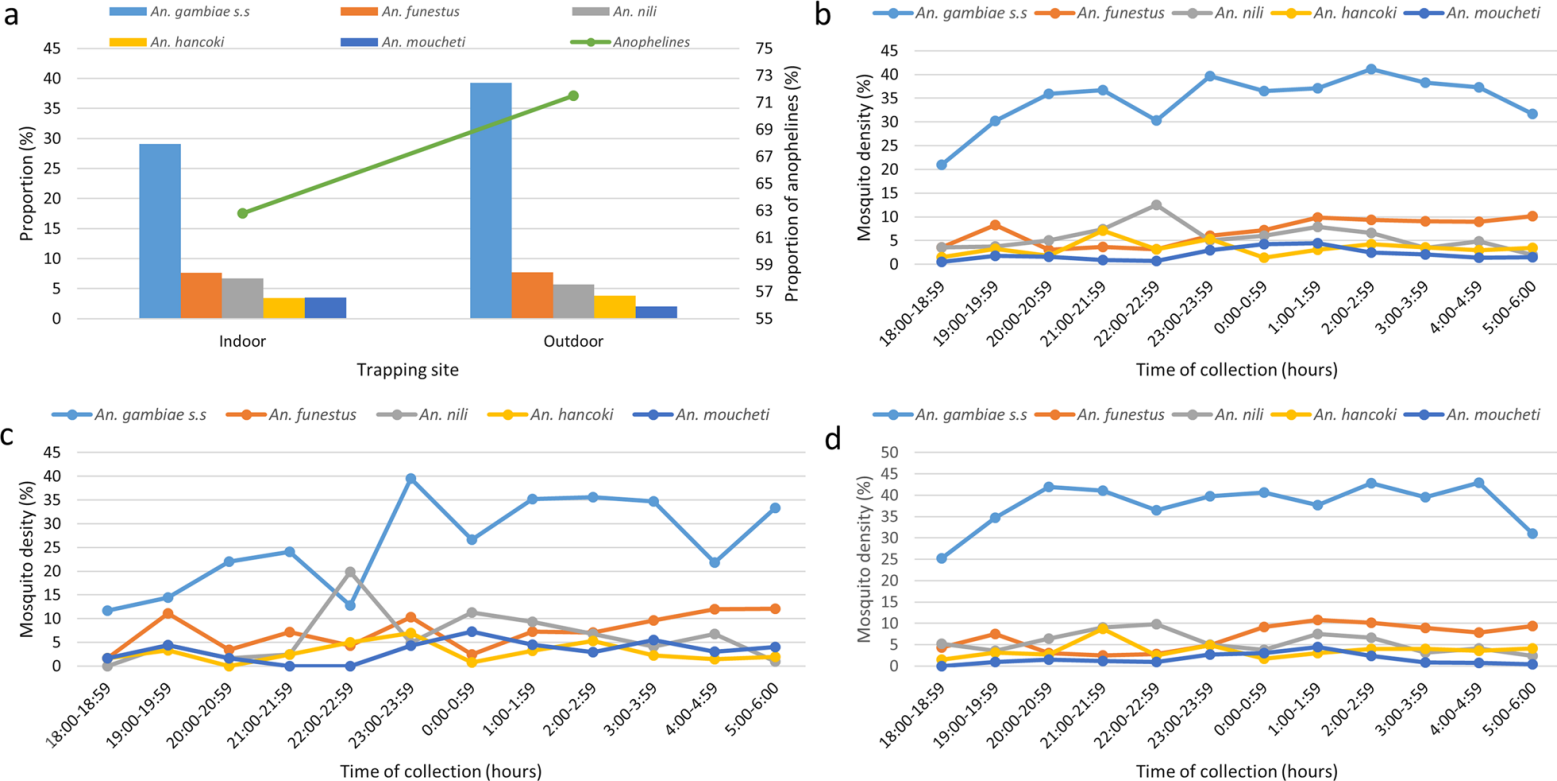
Outdoor and indoor exposure of residents to host-seeking anophelines and biting behavior. **a** Anopheline density at different trapping sites. **b** Overall biting behavior. **c** Indoor biting behavior. **d** Outdoor biting behavior

The HLC collections indicated outdoor and indoor exposure of residents to host-seeking anophelines (Fig. 4). In this survey, there was a significant association between the trapping site and the mosquito genera (*χ*^2^ = 39.29, *df* = 4, *P* < 0.001) and major malaria vector species (*χ*^2^ = 52.58, *df* = 4, *P* < 0.001). Most (71.5%, 3924/5488) of the *Anopheles* species were caught outdoors (Fig. 4), indicative of the strongly exophagic biting habits of the anophelines. A greater proportion of all major vectors were trapped outdoors except for *An. nili* and *An. moucheti*, which were more abundant indoors. Most anoplelines were trapped outdoors in the rainy season (*χ*^2^ = 43.47, *df* = 4, *P* < 0.001) as well as at low (*χ*^2^ = 24.09, *df* = 4, *P* < 0.001) and intermediate (*χ*^2^ = 29.18, *df* = 3, *P* < 0.001) altitudes. However, most *Anopheles* spp. were trapped indoors in the dry season (*χ*^2^ = 46.11, *df* = 3, *P* < 0.001) and at high altitude (*χ*^2^ = 12.85, *df* = 3, *P* = 0.005). There was also a significant association between the major malaria vector species and the trapping site at low (*χ*^2^ = 15.67, *df* = 4, *P* = 0.003), intermediate (*χ*^2^ = 29.72, *df* = 4, *P* < 0.001) and high (*χ*^2^ = 8.37, *df* = 2, *P* = 0.015) altitude as well as in the dry (*χ*^2^ = 57.91, *df* = 4, *P* < 0.001) and rainy (*χ*^2^ = 16.58, *df* = 4, *P* = 0.002) seasons.

In the dry season at low and intermediate altitudes, there was a significant association between the proportion of the major malaria vectors and the site of collection, with all except *An. gambiae* complex trapped indoors. No major malaria vector was caught at high altitude during the dry season, although very few anophelines (73) were trapped, mostly outdoors (62), during the survey. The major vector population also varied significantly with the site of collection during the dry season, with all mosquitoes except *An. gambiae* complex at low (*χ*^2^ = 33.01, *df* = 4, *P* < 0.001) and intermediate (*χ*^2^ = 29.11, *df* = 4, *P* < 0.001) altitude caught indoors.

Of the very few major malaria vectors (27) trapped at high altitude during the rainy season, most (12) of the *An. gambiae* complex (16) as well as the only *An. hancocki* were caught outdoors. Nevertheless, eight of the ten *An. funestus* were trapped indoors at high altitude in the rainy season. The biting cycle of *Anopheles* species overall as well as both indoors and outdoors was slightly similar for the major malaria vectors. The overall biting peaks for *An. gambiae* complex, *An. funestus*, *An. nili*, *An. hancoki* and *An. moucheti* were at 2–3 a.m., 5–6 a.m., 10–11 p.m., 9–10 p.m. and 1–2 a.m. respectively. However, the biting activities of *An. gambiae* complex were slightly different indoors and outdoors as its peak biting period was at 11 p.m. and 2–3 a.m./4–5 a.m. indoors and outdoors respectively (Fig. 4).

### Infection and entomological inoculation rates

Fifty-two (16.6%) of the 313 anophelines tested for *P. falciparum* circumsporozoite antigen by ELISA at intermediate and low altitudes in both the rainy and dry season were infected (Table 2). The overall average infection rate was 20.2% (42/208) and 9.5 (10/105) during the rainy and dry seasons respectively. Of these, *An. funestus* was the most infectious in the area overall (28.1%, 16/57), with higher rates in the rainy (30.4%, 14/46) compared to the dry (18.2%, 2/11) season. Apart from the *An. hancocki*, which was not infectious in the dry season (*n* = 12), all the other anophelines carried *P. falciparum* circumsporozoite antigen, with 24 (16.4%) of the tested *An. gambiae* (s.s.) seropositive overall (Table 2). The infectivity rate of the vectors was higher overall at low (19.7%, 37/188) compared to intermediate altitude (12.0%, 15/125) and in the rainy compared to the dry season at low altitude (23.5%, 32/136 vs. 9.6%, 5/52) and at intermediate altitude (13.9%, 10/72 vs. 9.4%, 5/53).

**Table 2:** Infection and entomological inoculation rates of major anophelines in the Mount Cameroon region

Altitude	Mosquito species	Season of collection						Total		
		Rainy			Dry					
		SI	HBR	EIR	SI	HBR	EIR	SI	HBR	EIR
Low	*An. gambiae* (s.s.)	0.3	6.3	1.6	0.1	2.4	0.2	0.2	8.8	1.6
	*An. funestus*	0.3	1.0	0.3	0.2	0.5	0.1	0.3	1.5	0.4
	*An. nili*	0.2	0.7	0.2	0	0.3	0	0.2	1.0	0.2
	*An. hancocki*	0.1	0.8	0.0	0	0.2	0	0.1	1.0	0.1
	Total	0.2	8.8	2.1	0.1	3.4	0.3	0.2	12.2	2.5
Intermediate	*An. gambiae* (s.s.)	0.2	5.4	0.9	0.1	2.3	0.3	0.2	7.7	1.2
	*An. funestus*	0.3	1.5	0.4	0	0.5	0	0.3	2.0	0.5
	*An. nili*	0.1	0.9	0.1	0.1	0.3	0.0	0.1	1.2	0.1
	*An. hancocki*	0	0.4	0	0	0.3	0	0	0.7	0
	Total	0.1	8.1	1.1	0.1	3.5	0.3	0.1	11.6	1.4
Overall	*An. gambiae* (s.s.)	0.2	5.9	1.2	0.1	2.4	0.3	0.2	8.3	1.3
	*An. funestus*	0.3	1.2	0.4	0.2	0.5	0.1	0.3	1.7	0.5
	*An. nili*	0.2	1.1	0.2	0.1	0.3	0.0	0.2	1.4	0.2
	*An. hancocki*	0.0	0.6	0.0	0	0.2	0	0.0	0.8	0.0
	Total	0.2	8.8	1.8	0.1	3.4	0.3	0.2	12.2	2.1

*EIR* entomological inoculation rate; *HBR* human biting rate; *SI* sporozoite index

The human biting and entomological inoculation rates are shown in Table 2. The average EIR in the study area was 2.08 infective bites per person per night (ib/p/n), almost two fold higher at low (2.45 ib/p/n) than at intermediate altitude (1.39 ib/p/n) and five times higher during the rainy (1.76 ib/p/n) compared to the dry season (0.34 ib/p/n). *Anopheles gambiae* (s.s.) had the highest inoculation rates averaging 1.33ib/p/n for the two seasons and up to 1.58 ib/p/n in the rainy season at low altitude.

### Malaria parasite prevalence and *Anopheles* behavior

A total of 1233 participants in 6 communities were screened for *Plasmodium* spp. infection, the majority (990, 80.3%) of which were > 5 years of age. The overall prevalence of malaria parasite infection was 31.5%, higher in children at most 5 years of age (39.9%) compared to older participants (31.3%) (Table 3). The prevalence of malaria parasite infection was lowest in Mutengene and highest in Misellele, where almost half (48.6%) of the participants were shown to harbor the parasite. Incidentally, more than half of the children ≤ 5 years old in three out of the six communities (Misellele, Ombe and Tiko) were parasite positive (Table 3).

**Table 3:** Malaria parasite prevalence and *Anopheles* biting rate and parity status in different communities around the slopes of Mount Cameroon

Community	Altitude	N	Human* Plasmodium *spp. prevalence [% (n)]			*Anopheles biting*			Number dissected	Parous [% (n)]	
			Overall	≤ 5 years	> 5 years	Number mosquitoes trapped	Number trappers	Rate (bite/man/night)		Overall	During survey^a^
Dibanda	Intermediate	329	35.9 (118)	38.6 (32)	35.0 (86)	1952	96	20.3	620	58.4 (362)	67.1 (47)
Misellele	Low	105	48.6 (51)	77.8 (14)	42.5 (37)	298	48	6.2	93	76.3 (71)	71.4 (50)
Moliwe		229	30.6 (70)	26.1 (13)	32.5 (57)	908	96	9.5	82	63.2 (52)	41.4 (29)
Mutengene		322	18.9 (61)	15.1 (8)	19.7 (53)	517	96	5.4	180	57.8 (104)	68.6 (48)
Ombe		86	39.5 (34)	55.0 (11)	34.9 (23)	331	96	3.5	211	67.8 (143)	61.4 (43)
Tiko		162	33.3 (54)	55.9 (19)	37.2 (54)	681	96	7.1	138	62.3 (86)	70.0 (49)
Overall		1233	31.5 (388)	39.9 (97)	31.3 (310)	4687	528	8.8	1324	61.8 (818)	63.3 (266)

^a^Computed from a random set of 70 dissected *Anopheles* mosquitoes per community caught during the malaria parasite prevalence survey

**Fig. 5:**
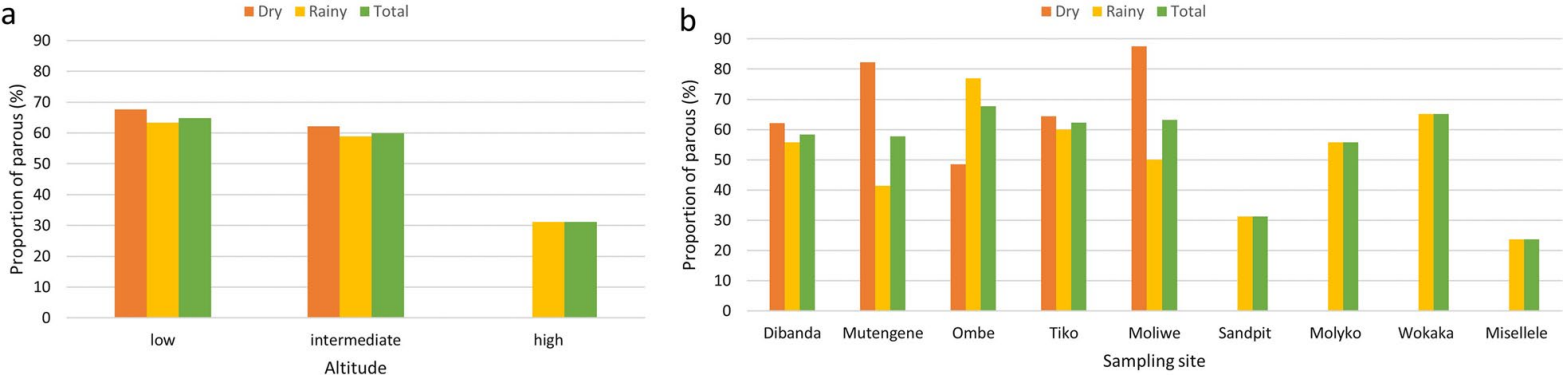
Variation in the proportion of parous anophelines in the rainy and dry season along the slopes of Mount Cameroon. **a** Variation with altitude. **b **Variation across sampling sites. No *Anopheles* species were caught at high altitude during the dry season

Of the 31.1% (1573/5065) randomly selected and dissected *Anopheles* species across all three altitudinal zones and both seasons, 971 (61.7%) were parous (Fig. 5). There was a significant variation in the parity status across different communities overall (*χ*^2^ = 23.84, *df* = 8, *P* = 0.002) as well as in the rainy (*χ*^2^ = 56.04, *df* = 8, *P* < 0.001) and dry (*χ*^2^ = 23.78, *df* = 4, *P* < 0.001) season. Although the overall proportions of parous anophelines in the dry [317 (64.8%)] and rainy [654 (60.3%)] seasons were similar (*χ*^2^ = 2.88, *df* = 1, *P* = 0.090), there was a significant association between parity status and altitude (*χ*^2^ = 10.30, *df* = 2, *P* = 0.006), highest at low altitude (447, 64.8%) and lowest at high altitude (5, 31.2%). Parity status was associated with altitude in the rainy season (*χ*^2^ = 7.80, *df* = 2, *P* = 0.020) but not in the dry season (*χ*^2^ = 1.62, *df* = 1, *P* = 0.203). There was no relationship between *Anopheles* biting or parity rate and malaria parasite prevalence in the selected communities for which both were simultaneously assessed (Table 3). Biting rates varied from 3.5 in Ombe to 20.3 bite/person/night in Dibanda while the proportion of parous *Anopheles* mosquitoes ranged from 41.4% in Moliwe to 71.4% in Misellele, where the prevalence of malaria parasite infection was highest (Table 3). All (100%, 5/5) *An. moucheti* dissected were parous compared to 65.6% (257/392), 64.3% (552/859) and 49.5% (156/315) of *An. funestus*, *An. gambiae* complex and *An. nili* respectively. Parity rate varied with altitude (*P* = 0.006) but not with season (*P* = 0.090) and site of collection (*P* = 0.990). The highest proportions of parous *An. gambiae* (83.9%, 26/31) were recorded between 8 and 9 p.m. while up to 81.8% (18/22) and 83.3% (60/72) of *An. funestus* between 7 and 8 p.m. and 1–2 a.m. respectively and 61.9% (13/21) and all three (100%) *An. nili* between 9 and 10 p.m. and 5–6 a.m. respectively were parous.

## Discussion

Malaria remains the leading cause of death to children < 5 years and pregnant women in most countries in SSA [1] with favourable malaria vectorial specificity and eco-climatic factors. Better understanding of the bio-ecology and spatiotemporal distribution of vectors is essential to design effective strategies for sustaining malaria control and elimination [32]. This study assessed key entomological parameters such as mosquito species composition, abundance, distribution and anopheline feeding behavior and biting activity in the rainy and dry season across different altitudinal zones 6 years following the intensification of vector control through the mass distribution of LLINs.

The study documents several different mosquito species and anopheline fauna around the forested slopes of Mount Cameroon, consistent with previous reports [9, 23, 24, 33]. The facts that *Anopheles* was the most abundant mosquito genera and several major *Anopeles* species were present all year round explain the high malaria burden in the region. The unequal distribution of the *Anopheles* species within the communities studied further confirms that mosquito occurrence is influenced by macro- and micro-environmental differences exhibited by different bio-ecological areas [24]. *Culex* spp. was the second most abundant mosquito genus in this study, in contrast to Amvongo-Adji et al. (2018) [9] who reported *Mansonia* as the second most common species. The increased density of Culicines, the primary host of several viruses, has implications for the transmission of West Nile fever, St. Louis encephalitis, Japanese encephalitis and lymphatic filariasis in the area.

The densities of both anophelines and culicines were greatly influenced by altitude and season, consistent with studies in the city of Yaoundé, Cameroon [34, 35], with the abundance of mosquitoes and anopheline fauna highest at low/intermediate altitudes and in the dry season. Previous studies around the slopes of Mount Cameroon have also reported more *Anopheles* species during the dry season [23, 36] compared to the rainy season, during which mosquito breeding is expected to be higher owing to increased rainfall and the presence of unwanted water bodies. This may accrue to the ever-enduring larval habitats in these villages but could also be explained by the ability of anopheline species to adapt and breed in several alternative water bodies. The Ndongo River that runs from Dibanda to Mutengene, for instance, is ever present while other streams run from Moliwe through Ombe, converging at the Tiko health district. The proportion of mosquitoes in the dry season may have been elevated by breeding sources left during the rainy season since some surveys were undertaken just at the end of the rains.

Consistent with previous reports [24], *A. gambiae* complex*, An. funestus, An. nili, An. hancoki* and *An. moucheti* were the first five major *Anopheles* species in decreasing order of prevalence. Of the anophelines whose densities were slightly lower than those of the major vectors, *An. cinctus*, *An. longipalpis*, *An. kingi*, *An. ziemanni* and *An. marshalli* are the proposed secondary vectors of malaria in the area. *Anopheles ziemanni* has been well characterized in several entomological studies as a major malaria vector in the northwestern region [37] or secondary vector elsewhere in the country [36] while this is the first report of *An. cinctus* as a possible secondary malaria vector in the southwestern part of the country.

Mosquito species of the *An. gambiae* complex are known to be heterogeneous in their biting behavior and could be endophilic, exophilic, anthropophilic or zoophagic, characteristics that enable the assessment of malaria transmission from vector biting rates. With up to 71.5% of the anopheline fauna caught outdoors, plenty of the *Anopheles* species still prefer resting outdoors as opposed to indoors as reported previously in the area and elsewhere [9, 24, 38]. Exophagic activity was the dominant behavior of the *An. gambiae* complex mosquitoes in this study, at a degree consistent with previous reports of mostly exophagous *An. gambiae* populations during the rainy season [6, 9]. However, *An. nili* and *An. moucheti* were mainly trapped indoors suggestive of a strongly endophagic behavior and contrary to previous studies [6, 9]. This modification in host-seeking behavior may have been induced by years of vector control through ITNs/LLINs and IRS and hence the need to change or augment these intervention strategies.

The overall biting activities of the major malaria vectors recorded in this study are consistent with others studies [9, 39], with members of the *An. gambiae* complex (2–3 am) and *An. funestus* group (5–6 am) biting late at night, between midnight and the early hours of the morning. This behavior clearly suggests increased biting activity when places are calm and the host is stable, coinciding with the resting periods (deep sleep) of their host. The slightly different indoor and outdoor biting peaks of *An. gambiae* complex may be due to the nocturnal habits of the hosts in the study area.

Most mosquitoes caught during both seasons were parous in accordance with previous studies [40]. In addition, most of the parous mosquitoes were trapped during the early evening hours. This coincides with the time *Anopheles* species start feeding and could be explained by the fact that the period corresponds to the time when an increased number of persons return home after diurnal activities. Overall, the transmission intensity was high, as shown by the parity rates, which also coincided with the high density of *An. gambiae* complex and other *Anopheles* species which are established as the primary malaria vector in the region and also in so many parts of Africa.

Multiple vectors are known to transmit malaria in the area [9, 23, 24], with the presence of *An. gambiae* complex together with *An. funestus and An. hancocki* at all altitudes as well as with *An. funestus and An. nili* at low and intermediate altitudes, consistent with the sympatric co-existence of these anophelines. However, the fact that only *An. gambiae* (s.s.) of the *An. gambiae* complex sibling species was identified following molecular characterization contradicts previous studies [9, 24]. It is possible that temporary rainfall-dependent larval habitats that could support breeding of *An. gambiae* (s.s.) [41] might have increased with increased amount of rainfall over the period, thus reducing potential *An. coluzzii* breeding sites [42].

All major vectors tested in this study were found to be infected by *P. falciparum,* with higher infectivity (and lower EIR) for *An. funestus* compared to *An. gambiae* (s.s.). *Anopheles gambiae* (s.s.) was clearly the most aggressive vector, with the higher EIR recorded, consistent with previous studies in the forested region of Cameroon [6, 23, 43]. Although higher infectivity of *An. funestus* relative to *An. gambiae* has been recorded previously in the area [44], this may accrue to the relatively fewer numbers of vector species trapped and tested during the survey, perhaps because of their low longevity, with most dying before the parasites can develop to infective stage [45, 46]. Also, higher EIRs obtained in rural areas were likely due to a high biting rate of *An. gambiae* (s.s.) *and An. funestus.* This should result from an exponential proliferation in breeding sites in rural settings, meeting optimum conditions for development, in contrast to urban areas where the larval habitats are generally quite polluted and therefore more conducive for the development of *Culicines* [47]. Transmission was shown to occur during both the dry and wet seasons, consistent with the perennial pattern in the area. Higher EIR in the rainy season and at lower altitude compared to the dry season and intermediate altitude is in line with previous reports of peak transmission during the heavy rains and at low altitude [21, 44]. Interventions such as IRS will thus be more impactful in the area if scheduled appropriately in line with varying transmission.

The fact that communities such as Misellele and Tiko with the highest malaria parasite prevalence in participants surveyed had the highest *Anopheles* fauna, biting and parity rates reaffirms the role of the vector in malaria morbidity. Targeted control interventions in some of these ‘hotspots’ may be indispensable in curbing the prevalence of the infection and incidence of disease in the area and region as a whole. However, the fact that almost half of the population of a community harbors the malaria parasite despite the tremendous and continuous deployment of LLINs, the main vector control strategy [48, 49] is concerning. This is because the massive scale up of LLIN, IRS and ITBN prevention tools between 2000 and 2016 resulted in substantial decrease of malaria morbidity and mortality across Africa (WHO 2018). It is possible that the emergence and rapid spread of malaria vectors resistant to pyrethroids, the most effective and rapidly acting single insecticide class used for impregnation of LLINs in the recent past [50] in several parts of Africa [51, 52] may have limited the impact of the intervention.

## Conclusion

*Anopheles gambiae* complex*, An. funestus, An. nili, An. hancoki* and *An. moucheti* were the most common *Anopheles* species around the slope of Mount Cameroon while *An. cinctus* was identified for the first time as a possible secondary vector for malaria transmission in the area. The biting cycles of *Anopheles* species overall as well as both indoors and outdoors were slightly similar for the major malaria vectors. Most *Anopheles* species across all three altitudinal zones and both seasons were parous, with the highest parity rate in communities with the highest proportion of individuals with malaria parasite infections. Regular monitoring of the bionomics of local Anopheles vector species and targeted control interventions in the ‘hotspots’ is necessary to curb the prevalence of the infection and the incidence of disease in the area and region as a whole.

## Data Availability

All data are provided within the manuscript.

## Abbreviations

CNERSH: Comite national D’ethique de la research pour la sante humaine
EIR: Entomological inoculation rate
ELISA: Enzyme-linked immunosorbent assay
HBR: Human biting rate
HLC: Human landing catches
ITN: Insecticide-treated nets
LLIN: Long lasting insecticide-treated nets
mRDT: Malaria rapid diagnostic test
OD: Optical density
PCR: Polymerase chain reaction
PR: Parity rate
SI: Sporozoite index
SSA: Sub-Saharan Africa
